# Lives Saved Tool (LiST) costing: a module to examine costs and prioritize interventions

**DOI:** 10.1186/s12889-017-4738-1

**Published:** 2017-11-07

**Authors:** Lori A. Bollinger, Rachel Sanders, William Winfrey, Adebiyi Adesina

**Affiliations:** Avenir Health, 655 Winding Brook Drive, Glastonbury, CT 06033 USA

**Keywords:** Lives saved tool, Costing, OneHealth Tool, LiST costing, LiST

## Abstract

**Background:**

Achieving the Sustainable Development Goals will require careful allocation of resources in order to achieve the highest impact. The Lives Saved Tool (LiST) has been used widely to calculate the impact of maternal, neonatal and child health (MNCH) interventions for program planning and multi-country estimation in several *Lancet* Series commissions. As use of the LiST model increases, many have expressed a desire to cost interventions within the model, in order to support budgeting and prioritization of interventions by countries. A limited LiST costing module was introduced several years ago, but with gaps in cost types. Updates to inputs have now been added to make the module fully functional for a range of uses.

**Methods:**

This paper builds on previous work that developed an initial version of the LiST costing module to provide costs for MNCH interventions using an ingredients-based costing approach. Here, we update in 2016 the previous econometric estimates from 2013 with newly-available data and also include above-facility level costs such as program management. The updated econometric estimates inform percentages of intervention-level costs for some direct costs and indirect costs. These estimates add to existing values for direct cost requirements for items such as drugs and supplies and required provider time which were already available in LiST Costing.

**Results:**

Results generated by the LiST costing module include costs for each intervention, as well as disaggregated costs by intervention including drug and supply costs, labor costs, other recurrent costs, capital costs, and above-service delivery costs. These results can be combined with mortality estimates to support prioritization of interventions by countries.

**Conclusions:**

The LiST costing module provides an option for countries to identify resource requirements for scaling up a maternal, neonatal, and child health program, and to examine the financial impact of different resource allocation strategies. It can be a useful tool for countries as they seek to identify the best investments for scarce resources. The purpose of the LiST model is to provide a tool to make resource allocation decisions in a strategic planning process through prioritizing interventions based on resulting impact on maternal and child mortality and morbidity.

**Electronic supplementary material:**

The online version of this article (10.1186/s12889-017-4738-1) contains supplementary material, which is available to authorized users.

## Background

Significant progress was made toward reaching the Millennium Development Goals (MDGs) for improved maternal, neonatal, and child health (MNCH), including reducing the number of child deaths globally from 12.7 million in 1990 to 6 million in 2015; reducing the number of underweight children from 28% of those under age 5 in 1990 to 14% in 2014; and reducing the number of maternal deaths from approximately 523,000 in 1990 to an estimated 303,000 in 2015 [1]. Although progress was steady, none of the MDGs were reached by 2015, resulting in their inclusion in various forms in the newly-agreed upon Sustainable Development Goals (SDGs), particularly SDG 3: Ensure healthy lives and promote well-being for all at all ages [2]. Achieving this SDG, along with all of the other SDGs, will require careful allocation of financial resources in order to achieve the highest impact. Utilizing tools to calculate the cost-effectiveness of targeted interventions can assist in the policy process of allocating scarce resources.

The Lives Saved Tool (LiST) has been used widely to calculate the impact of MNCH interventions [3–8], *in The Lancet* Series on Childhood Pneumonia and Diarrhoea [9] and in *The Lancet* Series on Maternal and Child Nutrition [10]. As use of LiST increases, many have expressed a desire to cost interventions within the model, in order to compare the costs and impact of a package of services. This paper builds on previous work that developed an initial version of the LiST costing module to provide costs for MNCH interventions using an ingredients-based costing approach [11]. Here, we update the previous econometric estimates with newly-available data and also include above-facility level costs such as program management. We utilize existing databases for drugs and supplies and provider time requirements, which, combined with the estimated costs for additional facility level overhead costs, provide users with a cost estimate at the program level for each intervention.

Results generated by the LiST costing module include total costs for each intervention, as well as disaggregated costs by intervention including drug and supply costs, labor costs, other recurrent costs, capital costs, and above-facility costs.

The LiST costing module is intended for use at the policy and strategic planning level, focusing on resource allocation decisions within MNCH programs. It can also be used to substantiate investment cases for MNCH and to support concept note development for the Global Financing Facility. It provides an estimate of the resources needed to implement an intervention or a package of services, but does not provide activity based costing, costing of program activities, or health systems strengthening requirements.

### Purpose of LiST costing

The purpose of LiST is to provide a tool for making resource allocation decisions in a strategic planning process by prioritizing interventions based on resulting impact on maternal and child mortality and morbidity; LiST Costing adds a cost dimension which provides a means to estimate resources required and the ability to compare costs for different packages of interventions. See Additional file 1 for a list of included interventions.

Questions can be explored such as: How much funding is required to achieve the goals of the strategic plan? What goals can be achieved with the current resources? What is the impact of alternative patterns of resource allocation in terms of both the associated costs and achieved goals of the strategic plan?

By using LiST Costing in conjunction with the standard LiST module, scenarios can be developed by varying parameters such as costs inputs, coverage rates of interventions, and/or other inputs, which can then be evaluated based on the impact on maternal and child mortality and morbidity and cost associated with delivering the package of services. Results from different scenarios can be displayed easily for comparison purposes. Applications can also be re-visited in subsequent years for evaluation purposes or can be updated to inform a new priority-setting exercise.

Impact analysis that addresses costs can be used to strengthen a country’s investment case for the Global Financing Facility or other donors, as well as contribute to discussions around domestic resource mobilization and identification of health program investment priorities. The results can be used beyond planning for the MNCH sector, including feeding into the development of a national health sector strategy, or even beyond the health sector to position the health budget within the broader government budget.

## Methods

The objective for developing the LiST costing module was to provide a means for estimating the financial cost of providing a service, while ensuring as much consistency as possible with data already available in LiST, as well as consistency with other methodologies followed by the WHO, such as the OneHealth Tool. Thus an econometric analysis was performed to estimate the contribution of intervention cost components that were not available in the OneHealth Tool. In addition, further methodological development of the LiST costing module was undertaken, including incorporating above-facility level costs into the existing intervention-level costs.

The impact estimates of LiST use coverage, effectiveness values and affected fractions to calculate mortality reductions or nutritional status improvements [10]. LiST costing builds on the following related concepts to estimate:

- *target population* - is the population on which the health intervention is focused, such as pregnant women or children aged <1 month;
- *population in need* – refers to the percentage of the target population that requires the intervention, such as the percent of pregnant women who need management of pre-eclampsia. For diseases such as diarrhea or malaria where there may be more than one case per year; this can be reflected in a percentage greater than 100%;
- *coverage* – refers to the *effective* coverage, i.e. the percentage of the population in need that actually receives the service
- *treatment inputs* – refers to drugs and supplies, medical personnel time requirements, and number of outpatient visits and inpatient days per case. Treatment inputs can be varied by delivery channel or level of service delivery, in order to reflect variation in drugs and supplies, skilled personnel, and other items that might be required when an intervention is delivered at higher levels of a health system.
- *costs per service* – calculated based on treatment inputs, and the unit costs for drugs and supplies, provider time, and costs of inpatient days and outpatient visits

In the LiST costing module, the final cost per service is calculated as the multiplication of all of these factors (calculations below are separated into two shorter equations):

Number of services = Target population * % population in need * coverage.

Cost per service (for each intervention) = Number of services * unit costs per service.

The total cost is the sum of all intervention costs, plus the costs of the above facility program costs.

Total costs = Sum of all costs per services + program costs.

**Fig. 1:**
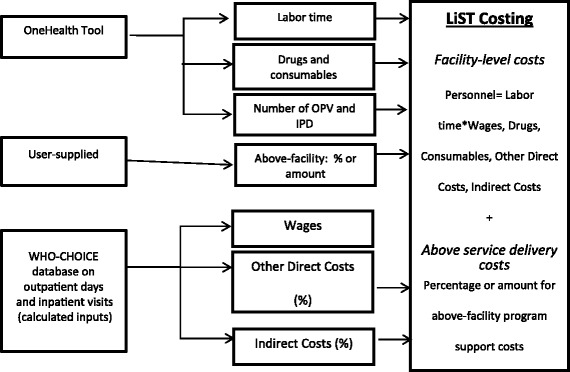
Schematic model and data sources for LiST costing

We define the full financial cost as the sum of facility-level and above-facility costs (see far right-hand column of Fig. 1).

### Intervention costs

Full facility-level unit costs are the sum of personnel costs (labor time * wages), drug and supply costs, other direct costs including nonconsumables and training that take place at the facility, and indirect costs including capital costs such as buildings, support personnel, maintenance/utilities, and supervision/management costs that are specific to the facility [12].

The number of minutes required to deliver a service by provider type is calculated based on the number of visits and time per visit specified in the treatment inputs for each intervention. A cost per minute for each provider type is calculated based on wages and benefits and time utilization.

Drug and supply costs are estimated using an ingredients approach, with information on the unit cost, percent of clients receiving, and number of units for each item (such as each dose of a vaccine, or drug, or supply such as a syringe).

Other recurrent and capital costs are applied as a cost per inpatient day and per outpatient visit. The number of inpatient days and visits are multiplied by the cost per day or visit.

### Program costs

Above-facility or program costs include all costs at the district, national, or international level that support the service, including program-level human resources, training that takes place above the facility level, supervision, monitoring and evaluation, infrastructure and equipment used in program-level activities, transport, communications, media and outreach, advocacy, and general program management. Each of these elements is explained further below; note that care should be taken to distinguish activities taking place at the facility vs. above-facility levels, especially in the case of training and capital costs, or double-counting could occur. Note further that all of these values can be changed in the software, and in an application should be reviewed and modified as appropriate.

For a pictorial representation of the LiST Costing cost items, see Fig. 1. As mentioned above, most default values for LiST Costing are derived from the OneHealth Tool or other WHO methodologies as much as possible when calculating the full financial cost of delivering a service. As shown in the top set of boxes in the two columns on the left-hand side of Fig. 1, we rely upon default values from OneHealth Tool for the quantity of labor (i.e., number of minutes spent delivering the service per cadre), as well as both the quantities and prices for drugs and for consumables used in LiST for each intervention. In addition, we also use the default values of the number of inpatient days (IPD) and outpatient visits (OPV) for each intervention. See the Data Sources section of this document for more information.

## Data sources

### Target populations and populations in need

Target populations are drawn from the demographic projection, the AIDS impact model and LiST, including numbers of pregnancies and births, and estimates of children in different age brackets. Populations in need are drawn from LiST inputs and results (including events such as pneumonia and sepsis, the percent of the population living in poverty, and nutritional status). Additional populations in need that are not available from a Spectrum impact model are drawn from OneHealth Tool databases, such as the percent of pregnant women needing management of pre-eclampsia.

### Treatment inputs: Drugs and supplies, provider time requirements, and outpatient visits and inpatient days

Treatment inputs for each intervention specify the required drugs and consumable supplies (e.g., gloves, syringes), provider time, and number of inpatient days and outpatient visits needed for the effective provision of an intervention. These are drawn from intervention assumptions developed for the OneHealth Tool and documented in an Input Intervention Assumptions Manual [13]. These inputs were developed based on WHO norms and guidelines where available, with expert input where explicit guidance was not available. Drugs and consumable supply prices are extracted from international sources such as the MSH International Medical Products Price Guide [14], UNICEF supply catalogue [15], and the Global Price Reporting Mechanism [16]. See Additional file 2 for more detail.

Default treatment inputs and prices are provided at a global level with no variation for different countries, but these inputs can be adjusted to fit the country context. Users can change the assumed quantities of drugs and supplies used, amount of provider time and number of visits, as well as the unit price for any input. In a usual country application, country teams review all data assumptions thoroughly, particularly country-specific input prices, and change the assumptions to reflect the specific country context.

Drug pricing defaults are updated every one to two years, depending on price stability for a given commodity. Updates for the drug prices that change more quickly, such as antiretroviral HIV/AIDS drugs, take place on an annual basis. Treatment inputs are updated when the treatment norms change, such as shifts in the drug regimens recommended by WHO, or following the introduction of new therapies. These changes typically take place less frequently and such database updates may occur every 3 years or longer intervals.

### Personnel costs

Default salaries are provided for each country based on WHO CHOICE [17], but can be changed by users if more relevant or updated information on salaries and benefits are available.

### Program costs

The user must supply either the amount of, or the percentage over and above, the facility-level costs associated with various above-facility program support activities, as shown in the second box on the left-hand side of Fig. 1. Above-facility program support activities include *program management*, or any activities above the facility level that are utilized in running the program at the district, national, or international level; *research*, which includes support for programs that collect primary data (such as national surveys, cohort studies, operations research, clinical trials), as well as data analysis, report writing, and dissemination; *monitoring and evaluation* (M&E), which includes salaries of M&E officers, equipment for data processing, communications, and transportation as well as the costs of collecting M&E data; *communication, media, and outreach*, which includes personnel costs for preparing analyses and presentations, conducting awareness-raising and policy dialogue with opinion leaders, policy makers, and stakeholders, office support costs, and transportation and materials production associated with dissemination activities; *training*, which includes both pre-service and in-service training for health care workers as well as special training for program staff in areas such as strategic planning, M&E, advocacy, and financial and reporting systems; and *infrastructure and equipment*, which includes costs associated with equipment acquired at the central, or above-facility, level. Many costs relating to improving the quality of care would be managed in this section. An initial suggested list of line items for program costs has been supplied by default but can be edited by the user.

### Other direct and indirect costs

The costs per outpatient visit and inpatient day (OPVs/IPDs) have been calculated by the World Health Organization at the country level and are available from the WHO-CHOICE website (http://www.who.int/choice/cost-effectiveness/en/). Note that the costs that are available on their website are defined by the World Health Organization to be the “hotel” cost portion of both OPVs and IPDs, that is, all costs except drugs and laboratory costs. In other words, the OPV and IPD costs contain both other direct costs (ODCs) and indirect costs, as well as personnel costs and the cost of consumables.

By estimating the proportion of the WHO-CHOICE OPV/IPD cost that is associated with ODCs and indirect costs, those proportions can then be applied to adjust the cost of the OPV/IPD for each intervention in LiST. To calculate the costs associated with ODCs and indirect costs for each LiST intervention, we can use the number of OPV/IPD for each intervention, already available in LiST, and multiply those quantities by the proportion of the country-specific cost of one OPV/IPD, available on the WHO-CHOICE website that is attributable to ODCs and indirect costs.

In order to calculate the proportion of the country-specific cost of one OPV/IPD that is attributable to ODCs and indirect costs, we utilized a dataset on costs of voluntary medical male circumcision (VMMC). Although it would have been ideal to utilize a dataset encompassing all MNCH interventions in LiST in order to perform this estimation, unfortunately such a dataset does not exist. Instead, we use the VMMC dataset which contains complete, consistent, and disaggregated facility-level data across multiple facilities and countries (Kenya, Namibia, South Africa, Tanzania, Uganda, and Zambia), and which has been used extensively and described in peer-reviewed literature [18–21]. The key implicit assumption here is that the labor utilization is comparable between VMMC and MNCH services.

We estimate a four-factor cost function comprised of Personnel, Consumables (excluding drugs and laboratory costs), Other Direct Costs (including nonconsumables and training), and Indirect Costs (including capital costs, support personnel, maintenance/utilities, and supervision/management costs). We excluded drugs and laboratory costs from the Consumables variable in the VMMC dataset, where they were initially included, in order to mirror the “hotel” cost definition of the OPV/IPD cost in the WHO-CHOICE dataset. We estimated a factor shares equation in Stata using a translog cost function, correcting the standard errors for heteroscedasticity using the Huber/White/sandwich estimator (see Additional file 3 for a complete derivation of the estimated equation and complete regression results). We estimated two equations, one for hospitals to be applied to IPD costs, and one for health centers to be applied to OPV costs. Both equations performed well, with adjusted R squared of 0.98 for the hospitals equation and 0.99 for the health centers equation, and highly significant F-statistics. We then calculated the percentage associated with each factor share in both equations by differentiating the estimated equation with respect to each factor input.

## Results

### Other direct and indirect costs

Results show that ODCs, which represent the sum of nonconsumables and training at the facility level, account for approximately 9.8% of the total unit cost at hospitals, while they account for about 8.5% of the total unit cost at health centers (see Table 1). Thus the levels appear somewhat similar between the two types of facilities. Indirect costs (the sum of capital costs, support personnel, maintenance/utilities and supervision/management costs), however, account for a larger share of overall unit cost at health centers compared to hospitals, 29.0% versus 19.5% respectively.

**Table 1:** Percentages associated with each factor in factor shares equation

	Personnel (%)	Consumables (% -excluding drugs and labs)	Other Direct Costs (%)	Indirect costs (%)
Hospitals	39.0%	31.7%	9.8%	19.5%
Health Centers	36.0%	23.4%	8.5%	29.0%

Authors’ calculations. Regression results from Stata using the VMMC cost dataset described in the text

### LiST costing

Results generated by the LiST costing module include total costs for each intervention, as well as disaggregated costs by intervention including drug and supply costs, labor costs, other recurrent costs, capital costs, and above-facility costs. Results can be written out to an Excel file for further manipulation, e.g., to perform cost-effectiveness analyses.

## Discussion

The results described above will provide a tool for users to estimate the costs of implementing a package of maternal and child health interventions, including costs at the above-facility level. The econometric estimates provide up-to-date coefficients for the proportion of the intervention-level unit costs that are due to ODCs and indirect costs of IPDs and OPVs. These results will also help user calculate cost-effectiveness ratios correctly. Costing done with List is an improvement over previous options, as it builds on the coverage estimates in LiST as well as target populations and populations in need that are dynamically updated as risk statuses, nutritional statuses and incidence are automatically updated through the epidemiology and demography calculations in Spectrum (including LiST, FamPlan, AIM, and Demproj).

### Links with impact calculations

LiST costing utilizes the coverage levels specified by users in the standard LiST editors. This coverage is utilized as part of the equation to establish number of services, as detailed in the methods section of this article. This ensures consistency between the cost and impact calculations of the tool.

### Comparing LiST costing to other costing tools

Several tools exist to facilitate strategic planning for MNCH programs, including LiST Costing, the OneHealth Tool, and EQUIST. Users should select from among them based on their analysis questions and purposes for the information being generated.

LiST costing is designed to help users prioritize from among a core set of MNCH interventions. It is automatically linked with LiST impact calculations and incorporates additional costing databases, such as wages, which allows for relatively quick generation of results to allow program planners and policy makers to think through the potential cost and resources required for implementing different packages of services.

The OneHealth Tool is designed for holistic health sector costing and budgeting and includes a broad suite of costing and impact analysis modules, including MNCH (impact calculated via LiST), but also HIV, TB, NCDs, malaria, and others. It requires more data collection and entry by the user around the health system costs such as wages, and infrastructure costs such as construction and operation of facilities. It provides detailed costing templates around the cost categories that are aggregated into other recurrent costs, capital cost, and program costs in LiST costing (including modules for human resources, infrastructure, logistics, and activity based program costing elements). This tool is typically used when estimating cost and impact of a whole health sector plan. Users who are focused more on MNCH interventions, and don’t need to have detailed outputs on health systems components will likely use LiST costing, while the OneHealth Tool will be preferred by users looking at a broader set of interventions and/or health systems implications of a plan.

EQUIST is a web-based tool designed to help policy makers identify strategies and approaches to reduce health disparities between advantaged and disadvantaged groups (defined via wealth, region, or urban or rural residence), and to examine the health outcomes of achieving greater parity. For more detail on different tools which include MNCH costing, see Table 2.

**Table 2:** Comparison of MNCH costing tools

	LiST Costing	OneHealth Tool	Equist (Equitable Strategies to Save Lives)
Main use	Resource allocation decisions for MNCH program	Broad health sector planning; cost and impact analysis for health plans and budgets.	Decision making to reduce mother and child health inequities
Costing methodology	Ingredients-based for interventions	Ingredients or activity based for interventions, program, and health systems costs	Incremental costing based on Marginal Budgeting for Bottlenecks methods
Disease scope	Maternal, neonatal, and child health	Sector-wide: RMNCH, HIV, malaria TB, NCDs, user-options to add other areas	Maternal, neonatal, and child health
User inputs	User must enter coverage targets, either numeric or percentage estimates for program costs; has the option to edit most other entries including treatment inputs and wage information	User must enter coverage targets, program costs, and health system specifications; treatment inputs editable as needed	User designs strategies for reducing inequities in order to view health outcomes and associated costs. Equist calculates coverages based on strategies.
Access	Free access; open download	Free access; open download	Open access with registration
Users	National, local and international planners, researchers, MOH, MNCH programs	Policy and planning departments; program managers	Programmers and planners
Time for implementation	1–4 weeks, depending on level of customization	3–6 months	Varies by country
Software	Spectrum software in Windows	Spectrum software in Windows; data can be copied from other software e.g., Excel.	Web based platform

Researchers and program planners should think through their research question and select the appropriate tool accordingly. Complementarity does exist between these tools: LiST costing is in many ways a streamlined and MNCH-focused component of the OneHealth Tool, and EQUIST uses LiST for its impact calculations. However, none are identical in their outputs and approach to analysis, so it is worth taking the time to think through the scope and specific goals which users are trying to achieve and select accordingly.

Limitations include using VMMC data in the estimation of ODCs and indirect cost proportions for a maternal and child health model; ideally a comprehensive, consistent dataset specific to MNCH would be utilized, but to our knowledge none are available.

Moving forward, one area of further development would be to facilitate the ease of regional applications. As health systems become more and more decentralized, increasingly planning will take place at a sub-national level. One suggestion is to provide a way to link a preset data input form directly with LiST, so that regional-level data can be utilized more easily.

## Conclusions

Achieving the Sustainable Development Goals requires choices to be made for investment in cost-effective interventions. Here, we describe the methods, data and results of the LiST Costing module which allows countries and researchers to calculate both the full financial costs and the impacts of MNCH intervention scale up on morbidity and mortality. It relies on existing intervention inputs and cost databases as much possible to ensure consistency with other models which countries may be using, while providing a more streamlined and simpler platform for estimates of program costs. As donors and policy makers consider the range of options available for health investment, evidence-based analysis of different resource allocation strategies is crucial, and LiST costing provides a user-friendly tool to consider cost effectiveness as a factor in these decisions.

## Additional files

Additional file 1: List of interventions included in LiST Costing. (DOCX 14 kb)

Additional file 2: Default drug and supply costs for selected services. (DOCX 17 kb)

Additional file 3: Cost functions. (DOCX 38 kb)

## Abbreviations

TB: Tuberculosis
IPD: Inpatient Days
LiST: Lives Saved Tool
M&E: Monitoring and Evaluation
MDGs: Millennium Development Goals
MNCH: Maternal, Neonatal and Child Health
NCD: Noncommunicable Diseases
ODCs: Other Direct Costs
OPV: Outpatient Visits
SDGs: Sustainable Development Goals
VMMC: Voluntary Medical Male Circumcision
