# Supplementary material for: Lives Saved Tool (LiST) costing: a module to examine costs and prioritize interventions
Source: BMC Public Health. 2017 Nov 7;17(Suppl 4):782. doi: 10.1186/s12889-017-4738-1 (PMC5688490; doi:10.1186/s12889-017-4738-1)
Supplement: Supplementary file 1 — List of interventions included in LiST Costing. (DOCX 14 kb) [file 12889_2017_4738_MOESM1_ESM.docx]

### Additional file 1 – Annex A

List of interventions included in LiST Costing

| Family planning | |
| --- | --- |
| Folic acid supplementation/fortification | |
| Safe abortion services | |
| Post abortion case management | |
| Ectopic pregnancy case management | |
| Blanket iron supplementation/fortification | |
| Pregnancy | |
| TT - Tetanus toxoid vaccination | |
| IPTp - Intermittent preventive treatment of malaria during pregnancy | |
| Syphilis detection and treatment | |
| Calcium supplementation | |
| Iron supplementation in pregnancy | |
| Multiple micronutrient supplementation in pregnancy | |
| Balanced energy supplementation | |
| Hypertensive disorder case management | |
| Diabetes case management | |
| Malaria case management | |
| MgSO4 management of pre-eclampsia | |
| Fetal growth restriction detection and management | |
| PMTCT - Prevention of mother to child transmission of HIV (including breastfeeding choices) | |
| Childbirth | |
| Clean birth practices | |
| Immediate assessment and stimulation | |
| Labor and delivery management | |
| Pre-referral management of labor complications | |
| Treatment of postpartum hemorrhage | |
| Management of obstructed labor | |
| Neonatal resuscitation | |
| Antenatal corticosteroids for preterm labor | |
| Antibiotics for pPRoM | |
| MgSO4 management of eclampsia | |
| AMTSL - Active management of the third stage of labor | |
| Induction of labor for pregnancies lasting 41+ weeks | |
| Promotion of breastfeeding | |
| Preventive | |
| Clean postnatal practices | |
| Chlorhexidine | |
| Feeding and supplements | |
| Complementary feeding - education only | |
| Complementary feeding - supplementary feeding and education | |
| Vitamin A supplementation | |
| Zinc supplementation | |
| WASH | |
| Improved water source | |
| Water connection in the home | |
| Improved sanitation - Utilization of latrines or toilets | |
| Hand washing with soap | |
| Hygienic disposal of children's stools | |
| ITN/IRS - Households protected from malaria | |
| Vaccines | |
| BCG vaccine | |
| Polio vaccine | |
| Pentavalent vaccine | |
| DPT vaccine | |
| H. influenzae b vaccine | |
| HepB vaccine | |
| Pneumococcal vaccine | |
| Rotavirus vaccine | |
| Measles vaccine | |
| N. meningitidis A vaccine | |
| Curative after birth | |
| Maternal sepsis case management | |
| Case management of premature babies | |
| Thermal care | |
| KMC - Kangaroo mother care | |
| Full supportive care for prematurity | |
| Case management of neonatal sepsis/pneumonia | |
| Oral antibiotics for neonatal sepsis/pneumonia | |
| Injectable antibiotics for neonatal sepsis/pneumonia | |
| Full supportive care for neonatal sepsis/pneumonia | |
| Diarrhea | |
| ORS - oral rehydration solution | |
| Antibiotics for treatment of dysentery | |
| Zinc for treatment of diarrhea | |
| Other infectious diseases | |
| Oral antibiotics for pneumonia | |
| Vitamin A for treatment of measles | |
| ACTs - Artemesinin compounds for treatment of malaria | |
| SAM - treatment for severe acute malnutrition | |
| MAM - treatment for moderate acute malnutrition | |
| HIV | |
