# Supplementary material for: Lives Saved Tool (LiST) costing: a module to examine costs and prioritize interventions
Source: BMC Public Health. 2017 Nov 7;17(Suppl 4):782. doi: 10.1186/s12889-017-4738-1 (PMC5688490; doi:10.1186/s12889-017-4738-1)
Supplement: Supplementary file 2 — Default drug and supply costs for selected services. (DOCX 17 kb) [file 12889_2017_4738_MOESM2_ESM.docx]

Additional file 2 – Default drug and supply costs for selected services

| Lives Saved Tool (LiST) - Treatment inputs - LiST costing |  |
| --- | --- |
|  | US$ |
| Family planning |  |
| Pill | $ 6.15 |
| Condom - Male | $ 7.86 |
| Injectable - 3 month (Depo Provera) | $ 3.42 |
| IUD - Copper-T 380-A IUD (10 years) | $ 1.27 |
| Implant - Jadelle (5 years) | $ 7.37 |
| Female sterilization | $ 4.67 |
| Male sterilization | $ 1.86 |
| Vaginal barrier method | $ 25.00 |
| Vaginal tablets | $ 10.00 |
| Folic acid supplementation/fortification | $ 0.07 |
| Safe abortion services | $ 1.26 |
| Post abortion case management | $ 15.60 |
| Ectopic pregnancy case management | $ 25.99 |
| Pregnancy |  |
| TT - Tetanus toxoid vaccination | $ 0.22 |
| IPTp - Intermittent preventive treatment of malaria during pregnancy | $ 0.06 |
| Syphilis detection and treatment | $ 0.55 |
| Nutritional |  |
| Calcium supplementation | $ 10.80 |
| Iron supplementation in pregnancy | $ 0.04 |
| Multiple micronutrient supplementation in pregnancy | $ 7.20 |
| Case management |  |
| Hypertensive disorder case management | $ 0.15 |
| Diabetes case management | $ 1.40 |
| Malaria case management | $ 2.55 |
| MgSO4 management of pre-eclampsia | $ 8.51 |
| HIV |  |
| PMTCT - Prevention of mother to child transmission of HIV | $ 22.02 |
| Childbirth |  |
| Clean birth practices | $ 1.37 |
| Immediate assessment and stimulation | $ 0.00 |
| Labor and delivery management | $ 2.26 |
| Pre-referral management of labor complications | $ 29.99 |
| Treatment of postpartum hemorrhage | $ 9.10 |
| Management of obstructed labor | $ 20.58 |
| Neonatal resuscitation | $ 0.38 |
| Antenatal corticosteroids for preterm labor | $ 3.40 |
| Antibiotics for pPRoM | $ 0.90 |
| MgSO4 management of eclampsia | $ 8.51 |
| AMTSL - Active management of the third stage of labor | $ 0.23 |
| Induction of labor for pregnancies lasting 41+ weeks | $ 0.00 |
| Postnatal care |  |
| Clean postnatal practices | $ 0.18 |
| Chlorhexidine | $ 0.42 |
| Feeding and supplements |  |
| Complementary feeding - supplementary feeding and education | $ 11.70 |
| Vitamin A supplementation | $ 0.10 |
| Zinc supplementation | $ 14.60 |
| ITN/IRS - Households protected from malaria | $ 1.50 |
| Vaccines |  |
| BCG vaccine | $ 0.22 |
| Polio vaccine | $ 0.25 |
| Pentavalent vaccine | $ 2.57 |
| DPT vaccine | $ 0.61 |
| H. influenzae b vaccine | $ 3.45 |
| HepB vaccine | $ 7.65 |
| Pneumococcal vaccine | $ 17.03 |
| Rotavirus vaccine | $ 2.53 |
| Measles vaccine | $ 0.35 |
| N. meningitidis A vaccine | $ 1.24 |
| Curative after birth |  |
| Maternal sepsis case management | $ 41.18 |
| Thermal care | $ 0.50 |
| Oral antibiotics for neonatal sepsis/pneumonia | $ 0.42 |
| Injectable antibiotics for neonatal sepsis/pneumonia | $ 1.24 |
| Full supportive care for neonatal sepsis/pneumonia | $ 1.96 |
| Diarrhea |  |
| ORS - oral rehydration solution | $ 0.24 |
| Antibiotics for treatment of dysentery | $ 0.12 |
| Zinc for treatment of diarrhea | $ 0.53 |
| Oral antibiotics for pneumonia | $ 0.16 |
| Vitamin A for treatment of measles | $ 0.13 |
| ACTs - Artemesinin compounds for treatment of malaria | $ 2.55 |
| SAM - treatment for severe acute malnutrition | $ 94.77 |
| MAM - treatment for moderate acute malnutrition | $ 44.09 |
| HIV |  |
| Cotrimoxazole | $ 9.76 |
| ART | $ 117.75 |
