# Supplementary material for: Lives Saved Tool (LiST) costing: a module to examine costs and prioritize interventions
Source: BMC Public Health. 2017 Nov 7;17(Suppl 4):782. doi: 10.1186/s12889-017-4738-1 (PMC5688490; doi:10.1186/s12889-017-4738-1)
Supplement: Supplementary file 3 — Cost functions. (DOCX 38 kb) [file 12889_2017_4738_MOESM3_ESM.docx]

### Additional file 3 – Annex C

Following Berndt and Wood [1] and Diewert [2], we assume a unit cost function exists that reflects a well-behaved production function consisting of four factors of production:

Equation C1: U = U(P, C, O, I)

where:

- U = unit cost,
- P = personnel unit cost,
- C = consumables unit cost,
- O = other direct unit cost (variable/recurrent costs excluding personnel and consumables cost), and
- I = indirect unit cost (capital/investment cost).

Applying Shepherd’s Lemma, and assuming that the cost function is also well-behaved, then

Equation C2: x_i_ (Q; p) = δU (Q; p) ; i = P, C, O, I

δp_i_

where x_i_ (Q; p) is the cost minimizing demand for input i needed to produce output Q≥0 given factor prices p»0.

Again following the literature we use a generalized approach to calculate the unit cost function through estimating a translog (transcendental logarithmic) cost function [1-3]. The four-input unit cost function from above can be written in natural logarithmic terms as follows, limiting the cross-terms to first-derivatives only:

Equation C2: ln U = ln α_0_ + α_P_ ln P + α_C_ ln C + α_O_ ln O + α_I_ ln I + β_PP_ ln P^2^

+ β_CC_ ln C^2^ + β_OO_ ln O^2^ + β_II_ ln I^2^ + β_PC_ ln P ln C + β_PO_ ln P ln O

+ β_PI_ ln P ln I + β_CO_ ln C ln O + β_CI_ ln C ln I + β_OI_ ln O ln I

where:

- U is the unit cost for VMMC,
- P, C, O and I are the personnel, consumables, other direct (variable or recurrent costs excluding personnel and consumables cost) and indirect (capital or investment) unit costs for a VMMC patient, respectively,
- α_0_ represents the intercept while α_P_, α_C_, α_O_ and α_I_ are the coefficients for the first-order terms,
- β_PP_, β_CC_, β_OO_ and β_II_ are the coefficients for the second-order terms, and
- β_PC_, β_PO_, β_PI_, β_CO_, β_CI_ and β_OI_ are the coefficients for the first-order cross terms.

Differentiating Equation C2 to derive factor shares results in the following equations:

Equation C3: Unit cost share of Personnel costs

δln U/ln P = α_P_ + 2 β_PP_ ln P + β_PC_ ln C + β_PO_ ln O + β_PI_ ln I

Equation C4: Unit cost share of Consumables costs

δln U/ln C = α_C_ + 2 β_CC_ ln C + β_CP_ ln P + β_CO_ ln O + β_CI_ ln I

Equation C5: Unit cost share of Other direct costs

δ ln U/ln O = α_O_ + 2 β_OO_ ln O + β_OP_ ln P + β_OC_ LN CO + β_OI_ ln I

Equation C6: Unit cost share of Indirect costs

δ ln U/ln I = α_I_ + 2 β_II_ ln I + β_IP_ ln P + β_IC_ ln C + β_IO_ ln O

The factor shares are constants that are determined by available technology [4] and represent the responsiveness of unit cost to changes in the levels of personnel, consumables, other direct and indirect cost such that:

- If α_P_  + α_C_ + α_O_ + α_I_ = 1, then the function has constant returns to scale,
- If α_P_  + α_C_ + α_O_ + α_I_ < 1, then the function has decreasing returns to scale, and
- If α_P_  + α_C_ + α_O_ + α_I_ > 1, then the function has increasing returns to scale.

The translog cost function was estimated using facility-level data to obtain estimates for a four-input unit cost symmetric function. In order to obtain the cost share for personnel, consumable, other direct and indirect costs, the translog function was differentiated with respect to each factor input as delineated in equations A3-A6 above evaluating the variables at their means (see Table C1).

Table C1: Descriptive statistics for regression variables: mean and standard deviations

|  | Mean | Standard deviation |
| --- | --- | --- |
| Ln(Hospital unit cost) | $ 3.58 | $ 0.58 |
| Ln(Personnel) | $ 2.54 | $ 0.78 |
| Ln(Consumables) | $ 2.17 | $ 0.54 |
| Ln(Other Direct Costs) | $ 0.42 | $ 1.96 |
| Ln(Indirect) | $ 1.32 | $ 1.51 |
|  |  |  |
| Ln(Health centre unit cost) | $ 3.43 | $ 0.54 |
| Ln(Personnel) | $ 2.28 | $ 0.92 |
| Ln(Consumables) | $ 1.88 | $ 0.95 |
| Ln(Other Direct Costs) | $ -.04 | $ 1.63 |
| Ln(Indirect) | $ 1.94 | $ 0.99 |

Table C2: Summary Output for Hospitals: Regression results from estimation of translog cost function with corrected standard errors

Table C3: Summary Output for Health Centres: Regression results from estimation of translog cost function with corrected standard errors

**References**

1. Berndt ER, Wood DO. Technology, Prices, and the Derived Demand for Energy. The Review of Economics and Statistics. 1975;57(3):259.

2. Diewert WE. Separability and a generalization of the Cobb-Douglas cost, production and indirect utility functions. Ottawa: Research Branch, Program Development Service, Dept. of Manpower and Immigration Dept. of Economics, University of British Columbia; 1973.

3. Kumbhakar SC. Modeling allocative inefficiency in a translog cost function and cost share equations: An exact relationship. Journal of Econometrics. 1997;76(1-2):351–6.

4. Deaton A, Muellbauer J. Economics and consumer behavior. Cambridge, MA; 1980.
